# 16/18 genotyping in triage of persistent human papillomavirus infections with negative cytology in the English cervical screening pilot

**DOI:** 10.1038/s41416-019-0547-x

**Published:** 2019-08-14

**Authors:** Matejka Rebolj, Adam R. Brentnall, Christopher Mathews, Karin Denton, Miles Holbrook, Tanya Levine, Alexandra Sargent, John Smith, John Tidy, Xenia Tyler, Henry Kitchener, on behalf of HPV Pilot Steering Group

**Affiliations:** 1 Cancer Prevention Group, School of Cancer and Pharmaceutical Sciences, Faculty of Life Sciences and Medicine, King’s College London, London, UK; 2 Centre for Cancer Prevention, Wolfson Institute of Preventive Medicine, Barts & The London School of Medicine and Dentistry, Queen Mary University of London, London, UK; 3 Severn Pathology, Southmead Hospital, North Bristol NHS Trust, Bristol, UK; 4 Clinical Virology, Manchester University NHS Foundation Trust, Manchester, UK; 5 Department of Cellular Pathology, Northwick Park Hospital, London, UK; 6 Cytology, Royal Hallamshire Hospital, Sheffield Teaching Hospitals NHS Foundation Trust, Sheffield, UK; 7 Department of Gynaecological Oncology, Royal Hallamshire Hospital, Sheffield Teaching Hospitals NHS Foundation Trust, Sheffield, UK; 8 Department of Cellular Pathology, Norfolk & Norwich University Hospitals NHS Foundation Trust, Norwich, UK; 9 Institute of Cancer Sciences, University of Manchester, St. Mary’s Hospital, Manchester, UK

**Keywords:** Cancer screening, Population screening

## Abstract

**Background:**

In the English pilot of primary cervical screening with high-risk human papillomavirus (HR-HPV), we exploited natural viral clearance over 24 months to minimise unnecessary referral of HR-HPV+ women with negative cytology. Three laboratories were permitted to use 16/18 genotyping to select women for referral at 12-month recall. We estimated the clinical impact of this early genotyping referral.

**Methods:**

The observed numbers of women referred to colposcopy and with detected high-grade cervical intraepithelial neoplasia (CIN2+), and of women who did not attend early recall in the three laboratories were compared with those estimated to represent a situation without an early genotyping referral. The 95% confidence intervals (CI) for the differences between the protocols were calculated by using a parametric bootstrap.

**Results:**

Amongst 127,238 screened women, 16,097 (13%) had HR-HPV infections. The genotyping protocol required 5.9% (95% CI: 4.4–7.7) additional colposcopies and led to a detection of 1.2% additional CIN2+ (95% CI: 0.6–2.0), while 2.3% (95% CI: 2.1–2.5) fewer HR-HPV+/cytology− women did not attend the early recall compared with the non-genotyping protocol.

**Conclusions:**

In a screening programme with high quality of triage cytology and high adherence to early recall,16/18 genotyping of persistent HPV infections does not substantially increase CIN2+ detection.

## Background

In England, the National Health Service (NHS) has provided cervical screening since 1988 through a “call and recall” Cervical Screening Programme (CSP). Women become eligible for screening at age 25 years. Thereafter, they are recalled for cytological screening every 3 years until age 50 years, and then five yearly until the age of 64 years. Nationwide roll-out of primary high-risk human papillomavirus (HR-HPV) screening triaged with cytology is planned to be implemented by the end of 2019. In 2013, a pilot of primary cervical screening with HR-HPV testing was set up in six large CSP laboratories, accounting for about 13% of the nationally screened population.^1^

The aim of substituting cytology with HR-HPV testing is to achieve greater sensitivity and increase screening intervals. Because of relatively poor specificity, however, reflex cytology is required to identify those HR-HPV+ women who require colposcopy referral. In several countries including the USA and Australia, HPV 16/18 genotyping is being used at baseline to identify women with negative cytology at increased risk of underlying cervical intraepithelial neoplasia (CIN), for immediate referral.^2,3^ In these cases, the decision to refer HPV 16/18 positive women is made on a single screening sample.

The English pilot also recognised a potential value of HR-HPV genotyping in triage, but it was considered that implementing it in the same way as those other countries, i.e. based on a single sample, would lead to an unsustainable increase in the demand for colposcopy. Hence, women with HR-HPV infections have been managed as shown in Table 1. At baseline and at 12-month early recall, the selection of HR-HPV positive women for colposcopy relied on positive cytology, defined as borderline change in squamous or endocervical cells or worse. This is equivalent to atypical squamous cells of undetermined significance (ASCUS, and atypical glandular cells of undetermined significance, AGUS, in the Bethesda 2014 classification), or worse. Evidence of 24-month persistence of a HR-HPV infection, regardless of concurrent cytology, also triggered referral for colposcopy. Additionally, three of the six laboratories used HPV 16/18 genotyping as a basis for more rapid referral for colposcopy in cases where there was persistent infection at the 12-month early recall in the absence of cytological abnormality. This means that a decision to refer cytology-negative women to colposcopy based on 16/18 genotyping is made only after two consecutive HR-HPV-positive samples.

**Table 1:** Management of women in the English pilot of primary cervical screening with HR-HPV testing

Time of testing	Genotyping triage	Non-genotyping triage^b^
Baseline test	HR-HPV-negative: routine recall at 3/5 years^a^ HR-HPV-positive/positive cytology: colposcopy HR-HPV-positive/negative cytology: early recall at 12 months	
Early recall at 12 months	HR-HPV-negative: routine recall at 3/5 years^a^ HR-HPV-positive/cytology positive: colposcopy	
	HPV 16/18-positive/cytology-negative: colposcopy Other HR-HPV-positive/cytology-negative: early recall at 24 months	HR-HPV-positive/cytology - negative: early recall at 24 months
Early recall at 24 months	HR-HPV-negative: routine recall at 3/5 years^a^ HR-HPV-positive: colposcopy	

^a^Depending on the woman’s age. The 3-year routine recall interval is used for women aged 25–49 years, whereas the 5-year interval is used for women aged 50–64 years.

^b^One of the laboratories recorded HR-HPV genotyping information using a DNA assay but did not use it for clinical management of HR-HPV-positive women

Both of these triage protocols were aimed at reducing the need for colposcopy by exploiting the substantial natural clearance rates of all HR-HPV infections, including HPV 16/18.^4–6^ The non-genotyping protocol with two early recalls within 24 months after screening, aimed to maximise the reduction in the need for colposcopy but was potentially vulnerable to the risk of non-adherence with an additional early recall. The genotyping protocol, expediting a referral of women with the most high-risk infections and reserving the second early recall for those whose infections are less likely to progress to cancer, aimed to reduce loss to follow-up at the second early recall and to maximise the detection of CIN2+ lesions requiring treatment. Hence, we evaluated the differences between the two protocols in the overall frequency of referral for colposcopy, detection of CIN2+ and CIN3+, and the loss to follow-up at early recall.

## Methods

### The pilot

The pilot started in May 2013 and the main outcomes have been described previously in detail.^7^ Briefly, six English CSP laboratories converted around a third of their screening population from primary liquid-based cytology (LBC) to primary HR-HPV screening. Conversion was population-based. The selection of administrative areas for conversion was not determined in a random process. Rather, the laboratories considered practical issues such as maintaining a single clinical management protocol in colposcopy practices serving each administrative area. During the pilot, the population age range and recommended screening intervals remained unchanged.

### Screening and diagnostic tests

Screening samples were taken within primary care and were collected in either SurePath (Becton Dickinson, Sparks, MD) or ThinPrep (Hologic, Marlborough, MA) LBC media. SurePath was used in three laboratories, while ThinPrep was used in the other three. In 2013–2014, two laboratories used Cobas 4800 (Roche, Rotkreuz, Switzerland, or Branchburg, NJ); two used RealTime (Abbott, Wiesbaden, Germany) and the remaining two used APTIMA (Hologic, Manchester, UK). Cobas and RealTime are HR-HPV DNA genotyping assays that report HPV 16 and HPV 18 separately from the 12 other HR-HPV genotypes, which are reported in combination. APTIMA is an HR-HPV mRNA assay detecting the 14 HR-HPV genotypes in combination.

All HR-HPV assays had previously been approved for primary screening within the CSP. Triage cytology was read under routine conditions with knowledge of a HR-HPV infection, and was quality controlled to CSP standards. Colposcopy was conducted according to national clinical practice guidelines. All diagnoses reflect routine cytopathology and histopathology in the CSP.

### Study design

The present study was designed to compare the outcomes of screening in the pilot with and without HPV 16/18 triage at the 12-month early recall. As the first screening invitation is sent at age 24.5 years, we included women aged 24–64 years at the time of the screening test. Additionally, women were included if they had been screened during the first (prevalence) round of primary screening with HR-HPV testing from the beginning of the pilot in May 2013 until December 2014 in the three Cobas or RealTime laboratories that used the HR-HPV genotyping information for the management of HR-HPV positive women (Table 1). Data on all subsequent tests and diagnoses were retrieved from the laboratories’ information systems until May 2017, which gave all women 29–49 months of follow-up after the primary screening test.

Women screened in the three laboratories that did not use HR-HPV genotyping information for the management of HR-HPV-positive women were not included as a comparator in this post hoc analysis. Two of these laboratories used the APTIMA assay. Unlike DNA assays that typically detect both transient infections and those integrated into a host’s genome, APTIMA has been designed to detect (predominantly) the latter type of infections. It has indeed been observed that this assay typically detects fewer HR-HPV infections than DNA assays, which ultimately leads to lower colposcopy rates in a routine screening programme.^8,9^ Consequently, using APTIMA data as a comparator would have introduced the effect of the assay’s different molecular target into the comparison of the triage protocols and hence could substantially affect analysis, particularly in terms of the number of colposcopies.

The prevalence screening episode for each woman was defined as starting with the first test recorded during the pilot period, i.e. the primary (baseline) test, and closed with any early recall tests or colposcopies. If the first recorded pilot test was preceded by another test within the two prior years, or if the test’s management code identified it as a follow-up to a recent cervical abnormality, the episode was excluded from further analysis. This is because those tests were unlikely to have been taken for the purpose of primary screening. Tests were linked using each women’s unique English NHS numbers.

In this analysis, the infecting HR-HPV genotype was determined at the primary test and remained fixed even if the genotype changed by the 12-month early recall. The effect of a genotype change on the studied outcomes was addressed in a sensitivity analysis (see below). Women were included in the 16/18 category regardless of any co-infecting genotypes.

Our primary endpoints were (1) the total number of colposcopies performed, (2) the number of HR-HPV-positive/cytology-negative women not adhering to early recall and (3) the number of detected CIN2+ lesions for each triage protocol. CIN2+ was chosen as one of the primary endpoints as this is the threshold for treatment, but the results are also presented for the more reproducible endpoint of CIN3 + .^10^

These outcomes were estimated based on aggregated observed data from the three genotyping laboratories (Table 2), and the following two sets of assumptions. Firstly, we assumed that all women would be referred as expected on the basis of their screening outcomes (Table 1). For a minority of women in the data where this did not happen (grey cells in Table 2), we assumed that they would have the same clinical outcomes as women who were referred as expected. As this was done consistently for both protocols, the calculated total numbers of colposcopies, CIN, and women not returning for early recall under the genotyping protocol differ slightly from those that were directly observed. Secondly, the 24-month outcomes in cytology-negative women persistently infected with HPV 16/18 at 12 months could not be directly observed for the non-genotyping protocol. We estimated them on the following assumptions: (a) that attendance at 24-month early recall and colposcopy would be the same as that observed among women infected with other HR-HPV genotypes, (b) that persistence of HR-HPV infections between the 12- and 24-month early recalls would be as that observed in a fourth pilot laboratory, which reported HR-HPV genotyping data but implemented a non-genotyping triage protocol (Table 1) and (c) that CIN2+ and CIN3+ prevalent at 12-month early recall would still be detectable at 24-month early recall, i.e. that there was no excess regression or progression between the two early recalls.^11^

**Table 2:** Observed outcomes for HR-HPV positive women in the three genotyping laboratories combined

	N	Yes	Unknown	Proportion Yes
Baseline				
HR-HPV+	127,238	16,097	258	12.7%
* Cytology* *+* *if HR-HPV* *+*	16,097	5287	0	32.8%
Had colposcopy if HR-HPV+/cytology+ after a record of referral	5287	5163	0	97.7%
PPV of colposcopy for CIN2+ if HR-HPV+/cytology+	5163	2135	0	41.4%
PPV of colposcopy for CIN3+ if HR-HPV+/cytology +	5163	1367	0	26.5%
Early recall at 12 months (HR-HPV+/cytology- at baseline)				
Had early recall testing after a record of referral	10,810	8964	125	83.9%
HR-HPV +	8964	5263	0	58.7%
* Cytology* *+* *if HR-HPV* *+*	5263	1410	23	26.8%
Had colposcopy if HR-HPV+/cytology+ after a record of referral	1410	1353	0	96.0%
PPV of colposcopy for CIN2+ if HR-HPV+/cytology+	1353	473	0	35.0%
PPV of colposcopy for CIN3+ if HR-HPV+/cytology+	1353	269	0	19.9%
* Cytology- if HR-HPV+*	5263	3830	23	72.8%
HPV 16 or 18+ if HR-HPV+/cytology−	3830	1072	0	28.0%
Had colposcopy if HPV 16 or 18+/cytology- after a record of referral	1072	789	233	94.0%
PPV of colposcopy for CIN2+ if HPV 16 or 18+ /cytology−	789	103	0	13.1%
PPV of colposcopy for CIN3+ if HPV 16 or 18+/cytology−	789	55	0	7.0%
Early recall at 24 months (HR-HPV other+/cytology− at baseline and HR-HPV+/cytology− at 12-month early recall)				
Had early recall testing after a record of referral	2758	2091	48	77.2%
* HR-HPV+*	2091	1368	0	65.4%
Had colposcopy after a record of referral	1368	1144	23	85.1%
PPV of colposcopy for CIN2+ if HR-HPV+	1144	117	0	10.2%
PPV of colposcopy for CIN3+if HR-HPV+	1144	56	0	4.9%
Early recall at 24 months (HPV 16 or 18+/cytology− at baseline and HR-HPV+/cytology− at 12-month early recall)^a^				
HR-HPV+	98	73	0	74.5%

Gray cells: Proportions of women who adhered to the type of clinical follow-up recommended by the protocol, calculated after exclusion of category “unknown” from the denominator (if non-zero). Where the “unknown” category was larger than zero, the value refers to women who had no record of referral to the type of follow-up that would be expected following the recommendations; for them, we assumed that their outcomes would be the same as the outcomes among women who had the correct record of referral. All other proportions are calculated using values in column “N” as the denominator, as there the “unknown” cells represent e.g. invalid testing outcomes (a normal occurrence in routine screening, which leads to tailored follow-up recommendations).

*CIN* cervical intraepithelial neoplasia, *HR-HPV* high-risk human papillomavirus, any of the 14 high-risk genotypes detectable by the Cobas and RealTime assays unless otherwise specified, *PPV* positive predictive value.

^a^Data from the fourth pilot laboratory, which recorded HR-HPV genotyping information using a DNA assay but did not use it for clinical management of HR-HPV positive women

Two sensitivity analyses were undertaken to assess the robustness of the findings. In the first of these, we addressed a subgroup of women with HPV 16/18 infections and persistently negative cytology at 12 months. Among these women, a relatively large proportion did not have a record of referral for colposcopy (Table 2). In the base case analysis, we assumed that this was at random. In the sensitivity analysis, we used two conventional extreme assumptions for parameters with uncertain true values, i.e. that (analysis S1a) all women in this subgroup would have attended colposcopy with CIN2+ detection doubled from the (observed) base case value; or (analysis S1b) only half of the women in this subgroup would attend with CIN2+ detection halved from the base case value. A lower CIN2+ detection could be expected, for example, in cases where HPV 16/18 infection had cleared by the 12-month early recall, but the woman remains HR-HPV-positive. Indeed, this situation represented about two-thirds of the women without a record of referral to colposcopy at 12 months in the observed data. In the second sensitivity analysis, persistence of infections between the 12- and 24-month early recalls in women with negative cytology and HPV 16/18 infections (which played a role in estimating the number of colposcopies in the non-genotyping protocol) was based on a small dataset from a single laboratory (*N* = 98). We varied the proportion of women with persistent infections as: (analysis S2a) the lower 5% confidence limit; or (analysis S2b) the upper 95% confidence limit.

### Statistical analysis methods

For both the number of colposcopies and the number of CIN2+ lesions detected, the relative difference was reported as the ratio between the absolute difference in the totals for the genotyping and the non-genotyping protocols (numerator) and the total number in the non-genotyping protocol (denominator). For the number of women not adhering to early recall, the total number with HR-HPV-positive cytology - negative samples at baseline was used as the denominator. The positive predictive value (PPV) of colposcopy for CIN2+ and CIN3+ was calculated using the number of women attending colposcopy as the denominator. Detailed formulae are reported in Supplementary information (Tables S1–3).

**Fig. 1:**
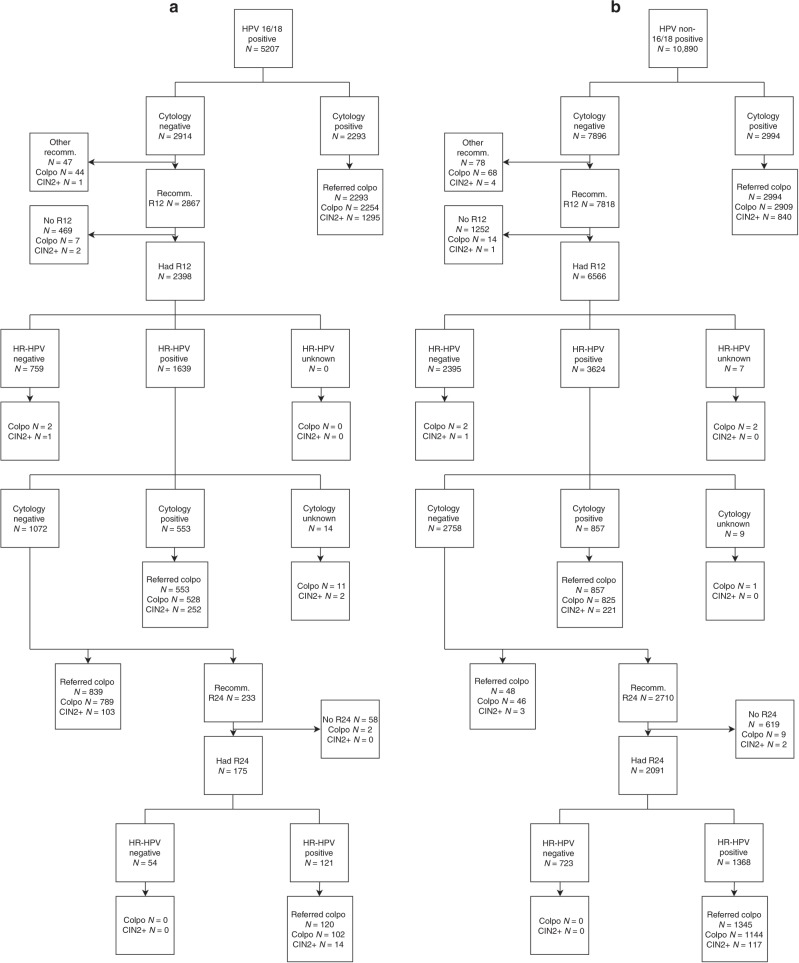
Screening outcomes including colposcopies and detection of CIN2+ outside of the recommended protocol. Screening was undertaken between May 2013 and December 2014, follow-up data were retrieved until May 2017. **a** Women with HPV 16/18 infections at baseline. **b** Women with HR-HPV infections other than HPV 16/18 at baseline. CIN cervical intraepithelial neoplasia, Colpo colposcopy, HPV human papillomavirus, R12 early recall at 12 months, R24 early recall at 24 months, Recomm. recommended

We obtained 95% confidence intervals (CI) for detection of CIN2+, number of colposcopies and loss of adherence to follow-up at the 12- and 24-month early recall using a parametric bootstrap. More precisely, following the flows in Fig. 1, we sampled the numbers in each category based on the observed data in Table 2; this process was repeated 10,000 times and the empirical distributions of the resulting numbers of colposcopies, CIN2+ and CIN3+, and women not attending early recall were used to form a 95% CI. The statistical software R (version 3.4.1) was used for all analysis.^12^

## Results

### Observed screening outcomes by HR-HPV genotype

In total, 127,238 women were screened in the three genotyping laboratories in 2013–2014. Of these, 16,097 (13%) had a positive HR-HPV test result, 5287 (4%) with positive and 10,810 (8%) with negative cytology (Table 2). In total, 8759 (7%) HR-HPV-positive women underwent a colposcopy, leading to detection of 2859 (2%) CIN2+ and 1763 (1%) CIN3+ (Table 3 and Fig. 1). These numbers include detection following the recommended management protocol, including early recall as well as any colposcopies undertaken outside the protocol. Colposcopies and CIN observed outside of the recommended protocol, for example those after an immediate referral of HR-HPV-positive cytology-negative women at baseline, were infrequent and were not included in further analyses. They amounted to 310 (4%) colposcopies, 31 (1%) CIN2+, and 16 (1%) CIN3+(Fig. 1).

**Table 3:** Observed distribution of HR-HPV infections and detected CIN2+, by HR-HPV genotype and the woman’s age

		Age group			
		24–29	30–49	50–64	Total
	N screened	23,864 (100%)	72,833 (100%)	30,541 (100%)	127,238 (100%)
	HR-HPV genotype at baseline				
HR-HPV infections	HR-HPV+	6709 (28%)	7646 (10%)	1742 (6%)	16,097 (13%)
	HPV 16+	2111 (9%)	1588 (2%)	348 (1%)	4047 (3%)
	Else HPV 18+	509 (2%)	541 (1%)	110 (<1%)	1160 (1%)
	Else other HR-HPV+	4089 (17%)	5517 (8%)	1284 (4%)	10,890 (9%)
Colposcopies	HR-HPV+	4013 (17%)	3890 (5%)	856 (3%)	8759 (7%)
	HPV 16+	1649 (7%)	1125 (2%)	215 (1%)	2989 (2%)
	Else HPV 18+	364 (2%)	325 (<1%)	61 (<1%)	750 (1%)
	Else other HR-HPV+	2000 (8%)	2440 (3%)	580 (2%)	5020 (4%)
CIN2+	HR-HPV+	1579 (7%)	1133 (2%)	147 (<1%)	2859 (2%)
	HPV 16+	899 (4%)	475 (1%)	49 (<1%)	1423 (1%)
	Else HPV 18+	138 (1%)	95 (<1%)	14 (<1%)	247 (<1%)
	Else other HR-HPV+	542 (2%)	563 (1%)	84 (<1%)	1189 (1%)
CIN3+	HR-HPV+	980 (4%)	699 (1%)	84 (<1%)	1763 (1%)
	HPV 16+	613 (3%)	324 (<1%)	31 (<1%)	968 (1%)
	Else HPV 18+	82 (<1%)	62 (<1%)	9 (<1%)	153 (<1%)
	Else other HR-HPV+	285 (1%)	313 (<1%)	44 (<1%)	642 (1%)

*CIN* cervical intraepithelial neoplasia, *HR-HPV* high-risk human papillomavirus

Detection of CIN2+ was highest among women screened at age of 24–29 years: 6.6% and 4.1% for CIN2+ and CIN3+, respectively. By comparison, the numbers were 1.6% and 1.0% at 30–49, and 0.5% and 0.3% at 50–64 years of age. A case of CIN2+ was detected for every three colposcopies at age of 24–29 years and for every six colposcopies at age of 50–64 years. For CIN3+, the numbers of colposcopies needed at these ages were four and 10 per case, respectively (data not tabulated).

Half of all CIN2+(50%, 1423/2859) and 55% (968/1763) of CIN3+ were diagnosed in women infected with HPV 16, whereas 9% (247/2859 and 153/1763) of CIN2+ and CIN3+ were detected in women with HPV 18 without HPV 16 (Table 3). Other genotypes without either HPV 16 or 18 were detected in 41% (1189/2859) of CIN2+ and 36% (642/1763) of CIN3+. Among all 4047 women infected with HPV 16, 35% (1423/4047) were ultimately diagnosed with CIN2+ and 24% (968/4047) with CIN3+. For the 1160 women infected with HPV 18, this was 21% (247/1160) and 13% (153/1160), respectively, and for the remaining 10,890 women with other HR-HPV infections it was 11% (1189/10,890) and 6% (642/10,890), respectively (Table 3).

During the same period, the fourth laboratory with HR-HPV DNA genotyping information, but implementing a non-genotyping triage protocol, screened 15,831 women with HR-HPV testing. Of these, 1714 (11%) had a positive HR-HPV test result, 1274 (8%) with negative and 440 (3%) with positive cytology. This was similar to the screening results in the three substantially larger laboratories included in the main analysis. Among the 98 women with HPV 16/18 infections and negative cytology persisting at 12 months, the infection persisted until 24 months in 73 (74%). This proportion was virtually constant across age groups (data not tabulated). Among women with HPV 16/18 infections who attended colposcopy after the 24-month early recall, the observed PPV for CIN2+ was 27% (19/71), with 15% (8/54) if they had negative cytology, and 65% (11/17) if they had positive cytology.

### Estimating the impact of the genotyping triage protocol

The genotyping protocol generated detection of 2869 CIN2+ and 1769 CIN3+ resulting from 8750 colposcopies among the 127,238 screened women (Table 4). More than 90% of all CIN2+ (91%, 2614/2869) were detected after a referral with positive cytology at either the baseline test or at the 12-month early recall. An additional 5% (133/2869) of CIN2+ were detected after a referral of HPV 16/18 positive women with persistently negative cytology at 12 months, and the final 4% (123/2869) of CIN2+ were diagnosed at 24-month early recall amongst women persistently infected with other HR-HPV genotypes. This pattern was very similar for the detection of CIN3+.

**Table 4:** Estimated numbers of colposcopies, high-grade CIN and women not attending the two early recalls following the genotyping and non-genotyping triage protocols, by time of testing

Time of testing	Screening test outcome at time of testing	HR-HPV genotyping protocol				Non-genotyping protocol			
		Colposcopies	CIN2+(PPV)	CIN3+(PPV)	Not attending early recall	Colposcopies	CIN2+(PPV)	CIN3+(PPV)	Not attending early recall
Baseline test	HR-HPV+ and cyt+	5163	2135 (41%)	1367 (26%)		5163	2135 (41%)	1367 (26%)	
Early recall at 12 months	Not attending early recall				1741				1741
	HR-HPV+ and cyt+	1369	479 (35%)	272 (20%)		1369	479 (35%)	272 (20%)	
	HPV 16/18+ and cyt-	1020	133 (13%)	71 (7%)					
Early recall at 24 months	Not attending early recall				637				864
	HPV+	1198	123 (10%)	59 (5%)		1728	221 (13%)	111 (6%)	
Total		8750	2869 (33%)	1769 (20%)	2378	8260	2835 (34%)	1751 (21%)	2626

*CIN* cervical intraepithelial neoplasia, *HR-HPV* high-risk human papillomavirus, *PPV* positive predictive value

An estimated 1741 cytology-negative women with a positive baseline HR-HPV test result did not attend the 12-month early recall. Additionally, 637 women who attended the 12-month early recall did not attend a recommended 24-month early recall. In total, we estimate that 22% (2378/10,810) of HR-HPV-positive cytology - negative women did not attend or complete early recall.

### Estimating the impact of the non-genotyping triage protocol

With this protocol, a total of 2835 CIN2+ and 1751 CIN3+ would be detected as a result of 8260 colposcopies among the 127,238 screened women (Table 4). Again, >90% of all high-grade CIN would be detected following positive triage cytology at baseline or at 12-month early recall. The remaining CIN2+ would be detected at the 24-month early recall for persistent HR-HPV.

Referring all persistently HR-HPV-positive women with negative cytology at 12 months to an additional 24-month early recall would result in 8% (864/10,810) of women not attending, in addition to the 16% (1741/10,810) not attending the 12-month early recall. In total, we estimate that 24% (2626/10,810) of HR-HPV-positive cytology-negative women would not have completed the recall under the non-genotyping triage protocol.

### PPV of a referral for colposcopy

The PPVs for CIN2+ were high when a colposcopy was undertaken following a positive cytology triage test result: 41% (2135/5163) at baseline and 35% (479/1369) after the 12-month early recall (Table 4).

In women infected with non-16/18 HR-HPV genotypes referred after the 24-month early recall, the PPV of a colposcopy was 10% (123/1198; Table 4). At this point, positive cytology was not used as a condition for a colposcopy. Nevertheless, the laboratories did report the cytology grade and the PPV for CIN2+ remained high, 29% (66/228), among women with cytological abnormalities, and much lower, 6% (51/907), among women who remained cytologically negative (data not tabulated; cytology of the remaining 9 out of 1144 women with a colposcopy (Table 2) was not graded).

In women with HPV 16/18 positive persistently negative cytology, the PPV for CIN2+ was 13% (133/1020) at the 12-month early recall. At 24 months, the PPV for persistent HPV 16/18 infections, regardless of cytology, is estimated at 18% ((221–123)/(1728-1198), Table 4). The PPV could not be reliably estimated separately by cytology but as reported earlier, it was 15% among 54 cytology - negative women in the fourth genotyping laboratory.

In all cases, the PPVs for CIN3+ were approximately half those for CIN2+.

### Comparison of the two protocols

We estimate that the genotyping protocol would detect an additional 34 (95% CI: 26–43) CIN2+ and 18 (95% CI: 13–24) CIN3+cases among the 127,238 screened women, representing 1.2% (95% CI: 0.9–1.5) of CIN2+ and 1.0% (95% CI: 0.8–1.4) of CIN3 + cases detectable by the non-genotyping protocol (Table 5). It would result in 5.9% (95% CI: 5.0–6.9) more colposcopies; 8750 (95% CI: 8572–8924) vs. 8260 (95% CI: 8079–8444), a difference of 490 (95% CI: 420–562). It would also result in 2.3% (95% CI: 2.1 to 2.5) fewer HR-HPV-positive cytology normal women not completing their recommended early recall; 2378 (95% CI: 2283–2475) vs. 2626 (95% CI: 2520–2731), a difference of 248 (95% CI: 226–270). The differences between the two protocols were very similar across all age groups (Table 5).

**Table 5:** Absolute and relative differences in the numbers of colposcopies, the numbers of detected CIN2+ and CIN3+ and in the numbers of women not attending early recall between the two triage protocols, by age at screening

	Absolute numbers per protocol							
	HR-HPV genotyping protocol				Non-genotyping protocol			
Age (years)	Colposcopies (95% CI)	CIN2 + (95% CI)	CIN3 + (95% CI)	Not attending early recall (95% CI)	Colposcopies (95% CI)	CIN2 + (95% CI)	CIN3 + (95% CI)	Not attending early recall (95% CI)
Total^a^	8750 (8572–8924)	2869 (2762–2973)	1769 (1686–1851)	2378 (2283–2475)	8260 (8079–8444)	2835 (2730–2937)	1751 (1668–1832)	2626 (2520–2731)
24–29	4003 (3889–4115)	1588 (1510–1663)	985 (924–1046)	1005 (945–1067)	3780 (3658–3900)	1566 (1489–1640)	973 (913–1033)	1137 (1069–1206)
30–49	3884 (3765–4000)	1135 (1068–1202)	701 (650–754)	1122 (1057–1187)	3665 (3543–3784)	1123 (1057–1189)	694 (644–747)	1221 (1150–1292)
50–64	862 (806–918)	147 (124–172)	84 (66–102)	252 (221–283)	810 (752–867)	146 (123–170)	83 (66–101)	276 (242–311)

	Differences between the protocols							
	Absolute differences (genotyping protocol—non-genotyping protocol)				Relative differences (vs. non-genotyping protocol)			
Age (years)	Colposcopies (95% CI)	CIN2 + (95% CI)	CIN3 + (95% CI)	Not attending early recall (95% CI)	Colposcopies (95% CI)	CIN2 + (95% CI)	CIN3 + (95% CI)	Not attending early recall (95% CI)^b^
Total^a^	+490 (+420 to +562)	+34 (+26 to +43)	+18 (+13 to +24)	−248 (−270 to −226)	+5.9% (+4.4 to +7.7)	+1.2% (+0.6 to +2.0)	+1.0% (+0.5 to +1.8)	−2.3% (−2.5 to −2.1)
24–29	+223 (+174 to +277)	+22 (+15 to +31)	+12 (+7 to +17)	−131 (−150 to −114)	+5.9% (+3.4 to +14.8)	+1.4% (+0.4 to +6.2)	+1.2% (+0.3 to +5.2)	−3.2% (−3.6 to −2.8)
30–49	+219 (+174 to +269)	+12 (+8 to +17)	+7 (+4 to +11)	−99 (−113 to −86)	+6.0% (+4.0 to +8.4)	+1.1% (+0.4 to +2.0)	+1.0% (+0.4 to +2.0)	−1.8% (−2.1 to −1.6)
50–64	+52 (+33 to +74)	+1 (+0 to +3)	+1 (+0 to +2)	−24 (−31 to −18)	+6.4% (+2.8 to +12.3)	+0.9% (+0.1 to +3.0)	+0.7% (+0.0 to +2.7)	−1.8% (−2.3 to −1.4)

^a^The totals as reported in Table 4. Sums by age differ slightly due to minor age-specific differences in completeness of follow-up and rounding

^b^Vs. the number of HR-HPV-positive cytology-negative women at baseline

The outcomes were not materially affected by varying the assumptions on the attendance at colposcopy and prevalence of CIN2+in HPV 16/18 positive women with persistently negative cytology. Under the favourable scenario for the genotyping protocol (analysis S1a: a high attendance at colposcopy and a high PPV), the latter would increase the need for colposcopy by 6.1% (95% CI: 5.2–7.0) and CIN2+ detection by 1.6% (95% CI: 1.3–1.9). Under the unfavourable scenario (analysis S1b: a low attendance at colposcopy and a low PPV), the estimates would be lower at 4.7% (95% CI: 3.8–5.6) and 0.3% (95% CI: 0.1–0.6), respectively. Varying the proportion of women infected with HPV 16/18 who remain HR-HPV-positive by 24 months produced a range in the extra demand for colposcopy between 6.6% (analysis S2a, 95% CI: 5.6–7.6) and 5.3% (analysis S2b, 95% CI: 4.5–6.1).

## Discussion

Using data from the English HPV pilot we estimated there would be a small increase in CIN2+ detection for HPV 16/18 genotyping compared with non-genotyping triage protocols for women with persistent HR-HPV infections and negative cytology. However, more rapid referral of persistently HPV 16/18 positive women with negative cytology would increase the number of colposcopies by 6%, which appears to be disproportionate with respect to an estimated increase in detected CIN2+ of 1%. This is a consequence of both reasonably high compliance with repeated testing in early recall observed in the pilot (close to 80%), and highly sensitive stratification of risk by cytology triage. The latter identified 75% of all CIN2+ at baseline and an additional 17% at 12-month early recall, with a high PPV on both occasions of over 30%. A very small pool of CIN2+ remained to be identified solely by HR-HPV genotyping but the PPV was substantially lower at around 10%.

As HPV 16/18 lesions are more likely to progress to cancer,^13–15^ our finding of a 1% higher detection of CIN2+ and CIN3+with a faster referral of HPV 16/18 positive women warrants consideration. This relatively small additional increase in the number of detected CIN2+ achieved by genotyping persistent HR-HPV infections would be observed on top of the ~50% increase achieved in the pilot by substituting cytology with HR-HPV testing,^7^ and most of these cases would be detected in women below 30 years of age, when the likelihood of regression of CIN2+ is highest.^16^ Persistently negative cytology is often associated with early infections and lesions detectable only through HR-HPV testing have been hypothesised to be small.^17^ Given the long duration of progression of CIN lesions to cervical cancer,^16,18,19^ a delay of 12 months in diagnosing these cytologically negative lesions is unlikely to be associated with a significant risk of interval cancer, provided women adhere to early recall.

HPV 16/18 genotyping has been recommended for an immediate referral of HR-HPV positive/cytologically negative women in countries such as the USA^2,20^ and Australia.^3^ In Europe, the attitude towards using genotyping in this manner has so far been more conservative,^21–23^ and baseline referral was not tested in the English pilot out of concern that it would lead to an unsustainable demand for colposcopy. When the switch was made from cytology to HR-HPV screening in the pilot, the demand for colposcopy increased by about 80% in the prevalence round.^7^ Had direct referral of all HPV 16/18 positive women been recommended, we estimate that referral would increase by an additional 15–20% (Supplementary information). As expected, viral clearance, however, was substantial (32% of women with HPV 16/18 infections and negative cytology tested HR-HPV negative at the 12-month early recall, and a further 26% tested negative at the 24-month recall). The immediate colposcopies in women destined to clear their infections are likely to have contributed to the very high average number of colposcopies needed to detect each CIN2 + case in the ATHENA study, which evaluated a setting with immediate colposcopy of all women aged ≥25 years with HPV 16/18 infections; this number was eight.^11^ In the English pilot, where cytologically negative women were only referred in the presence of a persistent infection, the number of colposcopies to detect a case of CIN2+ was three (8750/2869, Table 4).

Birth cohorts vaccinated against HPV 16/18 in the catch-up programme did not start entering the CSP until 2015, which means that our analysis is representative of an unvaccinated population. Through cross-protection, vaccination has the potential to decrease not only the prevalence of HPV 16/18 but also of certain other HR-HPV genotypes.^24^ As a result, the overall number of screened women who will require triage and colposcopy will decrease. The value of using genotyping for HPV 16/18 in the remaining persistent infections will probably decrease in line with the expected decrease in CIN2+ lesions associated with HPV 16/18.^25^

The large size and prospective protocol are key strengths of our study, as well as a population-based, routine HR-HPV-based screening setting using national standards and clinical guidelines, with quality assured HR-HPV testing, cytology, colposcopy and histology. The patterns of detection of CIN2+ by genotype (Table 3) were consistent with the literature. We were limited by having access to data from the laboratories participating in the pilot; if women moved away from the catchment areas of these six laboratories, their subsequent outcomes could not be traced. Nevertheless, the completeness of follow-up was high, about 95% after a referral for a colposcopy and about 80% after a referral for an early recall (Fig. 1). We could not directly observe the outcomes of a non-genotyping protocol. The resulting post hoc nature of our analysis required us to make several, albeit standard,^11^ assumptions on infection dynamics and the prevalence of CIN in women when managed following the non-genotyping protocol. Nonetheless, the sensitivity analyses showed that our conclusions were robust against a variety of assumptions. Additionally, using the data from the same three laboratories for both triage protocols meant that the background characteristics of the women, the catchment areas’ screening coverage and the cytology reading practices were constant. Finally, while our study compared two defined triage protocols, it cannot provide a conclusive answer as to what the optimal triage strategy would be for English HR-HPV positive women. A full optimisation study would require a substantially different approach comparing a number of alternative strategies, varying e.g. the eligibility criteria for triage, the number of early recalls, their timing, the tests and their positivity thresholds, and any age stratification.^26^ This is beyond the scope of our analysis.

## Conclusion

In population-based screening programmes with good quality of triage cytology and where most women adhere to early recall, HPV 16/18 triage of persistently HR-HPV-positive and cytologically negative women 12 months after primary screening can add very little in terms of a clinical benefit such as additional detection of CIN2+.

## Supplementary information

Supplementary material

## Data Availability

No additional unpublished data are available from the authors. Requests for access to data should be made to Public Health England, Office for Data Release.
