# Supplementary material for: 16/18 genotyping in triage of persistent human papillomavirus infections with negative cytology in the English cervical screening pilot
Source: Br J Cancer. 2019 Aug 14;121(6):455–63. doi: 10.1038/s41416-019-0547-x (PMC6738108; doi:10.1038/s41416-019-0547-x)
Supplement: Supplementary file 1 — Supplementary material [file 41416_2019_547_MOESM1_ESM.docx]

Cancer Prevention Group

School of Cancer & Pharmaceutical Sciences

Faculty of Life Sciences & Medicine

King’s College London

London, 19 June 2019

**16/18 GENOTYPING IN TRIAGE OF PERSISTENT**

**HUMAN PAPILLOMAVIRUS INFECTIONS WITH NEGATIVE CYTOLOGY IN THE ENGLISH CERVICAL SCREENING PILOT**

**SUPPLEMENTARY INFORMATION**

**Matejka Rebolj,^1§^ Adam R. Brentnall,^2^* Christopher Mathews,^1^***

**Karin Denton,^3^ Miles Holbrook,^4^ Tanya Levine,^5^ Alexandra Sargent,^4^**

**John Smith,^6^ John Tidy,^7^ Xenia Tyler,^8^ Henry Kitchener,^9^**

**on behalf of HPV Pilot Steering Group**

^1^ Cancer Prevention Group, School of Cancer and Pharmaceutical Sciences, Faculty of Life Sciences and Medicine, King’s College London, London, UK

^2^ Centre for Cancer Prevention, Wolfson Institute of Preventive Medicine, Barts & The London School of Medicine and Dentistry, Queen Mary University of London, London, UK

^3^ Severn Pathology, Southmead Hospital, North Bristol NHS Trust, Bristol, UK

^4^ Clinical Virology, Manchester University NHS Foundation Trust, Manchester

^5^ Department of Cellular Pathology, Northwick Park Hospital, London

^6^ Cytology, Royal Hallamshire Hospital, Sheffield Teaching Hospitals NHS Foundation Trust, Sheffield

^7^ Department of Gynaecological Oncology, Royal Hallamshire Hospital, Sheffield Teaching Hospitals NHS Foundation Trust, Sheffield

^8^ Department of Cellular Pathology, Norfolk & Norwich University Hospitals NHS Foundation Trust, Norwich

^9^ Institute of Cancer Sciences, University of Manchester, St. Mary's Hospital, Manchester, UK

* These authors contributed equally to this work

^§^Correspondence to:

Matejka Rebolj, Innovation Hub, Guy’s Cancer Centre, Great Maze Pond, London SE1 9RT, UK,

email: [matejka.rebolj@kcl.ac.uk](mailto:matejka.rebolj@kcl.ac.uk), phone: +44 (0)20 7848 4676

**SUPPLEMENTARY INFORMATION**

Estimation of the additional number of colposcopies in the case of an immediate referral of all HPV 16/18 positive women

Total number of colposcopies in the screened population (with the genotyping protocol): 8750 (Table 4)

Number of HPV 16/18 positive, cytology negative women at baseline: 2914 (Figure 1)

Attendance at colposcopy at baseline: 97.7% (observed for HR-HPV positive cytology positive women, Table 2)

Estimated needed number of colposcopies for direct referral of HPV 16/18 positive, cytology negative women: 2846 (0.977×2914)

Observed number of colposcopies in HPV 16/18 positive, cytology negative women (at any time during the early recall and including colposcopies outside of the recommended protocol): 1485 (Figure 1)

Difference between the estimated needed and the observed numbers: 1361 (2846-1485)

Relative increase in the number of colposcopies: 16% (1361/8750)

Estimation of the numbers of detected CIN, colposcopies, and women not attending early recall in the base case analysis

**Table S1.** Observed data. (Note: This is the same as Table 2 in the main text, with the addition of “Code” which simplifies the calculations below. “Unknown” results were excluded from the denominators in cases of referral that deviated from the recommended management protocol.)

| **Code** | **Description** | **Numerator** | **Denominator** | **Proportion** |
| --- | --- | --- | --- | --- |
|  | **BASELINE** |  |  |  |
| N | Number of women in the analysis | 127,328 | NR | NR |
| P1 | HR-HPV+ | 16,097 | 127,328 | 12.6% |
| P2 | Cytology+ if HR-HPV+ | 5287 | 16,097 | 32.8% |
| P3 | Had colposcopy if HR-HPV+/cytology+ after a record of referral | 5163 | 5287 | 97.7% |
| Q1 | PPV of colposcopy for CIN2+ if HR-HPV+/cytology+ | 2135 | 5163 | 41.4% |
| Q1b | PPV of colposcopy for CIN3+ if HR-HPV+/cytology+ | 1367 | 5163 | 26.5% |
|  | **EARLY RECALL AT 12 MONTHS (HR-HPV+/cytology- at baseline)** |  |  |  |
| P4 | Had testing at 12-month early recall after a record of referral | 8964 | 10,685 | 83.9% |
| P5 | HR-HPV+ | 5263 | 8964 | 58.7% |
| P6 | Cytology+ if HR-HPV+ | 1410 | 5263 | 26.8% |
| P7 | Had colposcopy if HR-HPV+/cytology+ after a record of referral | 1353 | 1410 | 96.0% |
| Q2 | PPV of colposcopy for CIN2+ if HR-HPV+/cytology+ | 473 | 1353 | 35.0% |
| Q2b | PPV of colposcopy for CIN3+ if HR-HPV+/cytology+ | 269 | 1353 | 19.9% |
| P6a | Cytology- if HR-HPV+ | 3830 | 5263 | 72.8% |
| P8 | HPV 16 or 18+ if HR-HPV+/cytology- | 1072 | 3830 | 28.0% |
| P9 | Had colposcopy if HPV 16 or 18+/cytology- after a record of referral | 789 | 839 | 94.0% |
| Q3 | PPV of colposcopy for CIN2+ if HPV 16 or 18+/cytology- | 103 | 789 | 13.1% |
| Q3b | PPV of colposcopy for CIN3+ if HPV 16 or 18+/cytology- | 55 | 789 | 7.0% |
|  | **EARLY RECALL AT 24 MONTHS (other HR-HPV +/cytology- at baseline and HR-HPV+/cytology- at 12-month early recall)** |  |  |  |
| P10 | Had testing at 24-month early recall after a record of referral | 2091 | 2710 | 77.2% |
| P11 | HR-HPV+ | 1368 | 2091 | 65.4% |
| P12 | Had colposcopy after a record of referral | 1144 | 1345 | 85.1% |
| Q4 | PPV of colposcopy for CIN2+ if HR-HPV+ | 117 | 1144 | 10.2% |
| Q4b | PPV of colposcopy for CIN3+ if HR-HPV+ | 56 | 1144 | 4.9% |
|  | **EARLY RECALL AT 24 MONTHS (HPV 16 or 18+/cytology- at baseline and HR-HPV+/cytology- at 12-month early recall)** |  |  |  |
| P14 | HR-HPV+ | 73 | 98 | 74.5% |

**Table S2. Genotyping protocol**. Women with HPV 16/18 infections who remain HR-HPV+/cyt- at 12-month early recall are referred to colposcopy. Women with other HR-HPV infections who remain HR-HPV+/cyt- at 12-month early recall are referred to 24-month early recall.

| **Time of testing** | **Screening test outcome at time of testing** | **Colposcopies** | **CIN2+** | **CIN3+** | **Not attending early recall** |
| --- | --- | --- | --- | --- | --- |
| **Baseline test** | **HR-HPV+/cytology+** | **N*P1*P2*P3=**  127328* (16097/127328)*  (5287/16097)* (5163/5287) = 5163 | **N*P1*P2*P3*Q1=**  5163* (2135/5163) = 2135 | **N*P1*P2*P3*Q1b=**  5163* (1367/5163) = 1367 |  |
| **Early recall at 12 months** | **Not attending** |  |  |  | **N*P1*(1-P2)*(1-P4)** =  127328* (16097/127328)* (1-(5287/16097))* (1-8964/10685) = 1741 |
|  | **HR-HPV+/cytology+** | **N*P1*(1-P2)*P4*P5*P6*P7**=  16097* (1-(5287/16097))* (8964/10685)* (5263/8964)* (1410/5263)* (1353/1410)= 1369 | **N*P1*(1-P2)*P4*P5*P6*P7*Q2=**  16097* (1-(5287/16097))* (8964/10685)* (5263/8964)* (1410/5263)* (1353/1410)* (473/1353)= 479 | **N*P1*(1-P2)*P4*P5*P6*P7*Q2b=**  16097* (1-(5287/16097))* (8964/10685)* (5263/8964)* (1410/5263)* (1353/1410)* (269/1353)= 272 |  |
|  | **HPV 16 or 18+/cytology-** | **N*P1*(1-P2)*P4*P5*P6a*P8*P9** =  16097* (1-(5287/16097))* (8964/10685)* (5263/8964)* (3830/5263)* (1072/3830)* (789/839) = 1020 | **N*P1*(1-P2)*P4*P5*P6a*P8*P9 * Q3 =** 16097* (1-(5287/16097))* (8964/10685)* (5263/8964)* (3830/5263)* (1072/3830)* (789/839) * (103/789) = 133 | **N*P1*(1-P2)*P4*P5*P6a*P8*P9 * Q3b =** 16097* (1-(5287/16097))* (8964/10685)* (5263/8964)* (3830/5263)* (1072/3830)* (789/839) * (55/789) = 71 |  |

**Table S2.** Continued.

| **Time of testing** | **Screening test outcome at time of testing** | **Colposcopies** | **CIN2+** | **CIN3+** | **Not attending early recall** |
| --- | --- | --- | --- | --- | --- |
| **Early recall at 24 months** | **Not attending** |  |  |  | **N*P1*(1-P2)*P4*P5*P6a*(1-P8)*(1-P10)** =  10810* (8964/10685)* (5263/8964)* (3830/5263)* (1-1072/3830)* (1-2091/2710) = 637 |
|  | **HR-HPV+** | **N*P1*(1-P2)*P4*P5*P6a*(1-P8)*P10*P11*P12**= 10810* (8964/10685)* (5263/8964)* (3830/5263)* (1-1072/3830)* 2091/2710 * (1368/2091) * (1144/1345) = 1198 | **N*P1*(1-P2)*P4*P5*P6a*(1-P8)*P10*P11*P12*Q4** = 10810* (8964/10685)* (5263/8964)* (3830/5263)* (1-1072/3830)* 2091/2710 * (1368/2091) * (1144/1345) * (117/1144) = 123 | **N*P1*(1-P2)*P4*P5*P6a*(1-P8)*P10*P11*P12*Q4b** = 10810* (8964/10685)* (5263/8964)* (3830/5263)* (1-1072/3830)* 2091/2710 * (1368/2091) * (1144/1345) * (56/1144) = 59 |  |
| **Total** |  | 8750 | 2870 | 1769 | 2378 |

**Table S3. Non-genotyping protocol.** All women with HR-HPV infections who remain HR-HPV+/cyt- at 12-month early recall are referred to 24-month early recall (regardless of genotype).

| **Time of testing** | **Screening test outcome at time of testing** | **Colposcopies** | **CIN2+** | **CIN3+** | **Not attending early recall** |
| --- | --- | --- | --- | --- | --- |
| **Baseline test** | **HR-HPV+/cytology+** | **N*P1*P2*P3=**  127328* (16097/127328)*  (5287/16097)* (5163/5287) = 5163 | **N*P1*P2*P3*Q1=**  5163* (2135/5163) = 2135 | **N*P1*P2*P3*Q1b=**  5163* (1367/5163) = 1367 |  |
| **Early recall at 12 months** | **Not attending** |  |  |  | **N*P1*(1-P2)*(1-P4)** =  127328* (16097/127328)* (1-(5287/16097))* (1-8964/10685) = 1741 |
|  | **HR-HPV+/cytology+** | **N*P1*(1-P2)*P4*P5*P6*P7**=  16097* (1-(5287/16097))* (8964/10685)* (5263/8964)* (1410/5263)* (1353/1410)= 1369 | **N*P1*(1-P2)*P4*P5*P6*P7*Q2=**  16097* (1-(5287/16097))* (8964/10685)* (5263/8964)* (1410/5263)* (1353/1410)* (473/1353)= 479 | **N*P1*(1-P2)*P4*P5*P6*P7*Q2b=**  16097* (1-(5287/16097))* (8964/10685)* (5263/8964)* (1410/5263)* (1353/1410)* (269/1353)= 272 |  |
| **Early recall at 24 months** | **Not attending** |  |  |  | **N*P1*(1-P2)*P4*P5*P6a*(1-P10)** = 10810* (8964/10685)* (5263/8964)* (3830/5263)* (1-(2091/2710)) = 885 |

**Table S3**. Continued.

| **Time of testing** | **Screening test outcome at time of testing** | **Colposcopies** | **CIN2+** | **CIN3+** | **Not attending early recall** |
| --- | --- | --- | --- | --- | --- |
|  | **HR-HPV+** | **N*P1*(1-P2)*P4*P5*P6a*P10*((1-P8)*P11+P8*P14)*P12** = 10810* (8964/10685)* (5263/8964)* (3830/5263)* (2091/2710)* ((1-(1072/3830))* (1368/2091) + (1072/3830)* (73/98))* 1144/1345 = 1728  *HR-HPV other: 1198*  *HPV 16/18: 530* | **N*P1*(1-P2)*P4*P5*P6a*(1-P8)*P10*P11*P12*Q4**  **+ N*P1*(1-P2)*P4*P5*P6a*P8* P10*Q3* (1/P9)* P12**= 10810* (8964/10685)* (5263/8964)* (3830/5263)* (1-1072/3830)* 2091/2710 * (1368/2091) * (1144/1345)* (117/1144)  + 10810* (8964/10685)* (5263/8964)* (3830/5263)* (1072/3830)* (2091/2710)* (103/789) * (839/789) * (1144/1345)= 221  *HR-HPV other: 1198*(117/1144)=123*  *HPV 16/18: 1085*(103/789)* (839/789)*(1144/1345) *(2091/2710)= 99* | **N*P1*(1-P2)*P4*P5*P6a*(1-P8)*P10*P11*P12*Q4b**  **+ N*P1*(1-P2)*P4*P5*P6a*P8* P10*Q3b* (1/P9)* P12**= 10810* (8964/10685)* (5263/8964)* (3830/5263)* (1-1072/3830)* 2091/2710 * (1368/2091) * (1144/1345)* (56/1144)  + 10810* (8964/10685)* (5263/8964)* (3830/5263)* (1072/3830)* (2091/2710)* (55/789) * (839/789) * (1144/1345)= 111  *HR-HPV other: 1198*(56/1144)=59*  *HPV 16/18: 1084*(55/789)* (839/789)*(1144/1345) *(2091/2710) = 53* |  |
| **Total** |  | 8260 | 2835 | 1750 | 2626 |
